# Is It Homœopathy or Isopathy?

**Published:** 1891-11

**Authors:** Samuel Swan

**Affiliations:** New York


### IS IT HOMŒOPATHY OR ISOPATHY?

SAMUEL SWAN, M. D., NEW YORK.

One great objection to the use of morbose products in the same disease which produced them is the idea they are isopathic, and many good and earnest physicians recoil with horror from such heretical therapeutics.

They cannot realize the change that potentization makes in the drug, changing it from an isopathic substance to a homœopathic remedy. " But," they say, " even if it is changed, Isopathy never cures, and though a morbose product may cure other persons, they never can cure the person from whom it was taken of the same disease."

This was thrown at me by a most excellent physician who ought to know better, and as he is proof against argument and reason, I send you the two cases below that entirely substantiate my position.

### ERYSIPELAS.

Mrs X. Caused by exposure to a very cold wind after having been to a concert in a very warm room. The next day there was marked evidence of fever, which increased steadily into and through the following night. The second day an erysipelatous swelling on the left cheek-bone appeared, extending toward the nose and back to the ear. At this juncture a physician was called who prescribed remedy after remedy without in any way checking the advance of the disease, which extended all over the face, forehead, and completely over the head. The eyes were closed, and it was difficult to distinguish the protuberance of the nose. The face was covered with immense blebs filled with a yellow serum. At this stage a second physician was called in, the first having became apprehensive of a fatal result.

At that time a quantity of the contents of the blebs with crusts, pus, and blood was put in a vial and covered with alcohol, and forwarded to Dr. Swan for potentization, with the request to return the potency as speedily as possible. The CMM was returned by next mail, and was at once given dry on the tongue, a powder every two hours until four doses had been taken. Before the last powder was given, the patient recognized a very marked relief in her condition of feelings. The first symptom noted after taking medicine, patient got cross and complained about everything. The day following, the change in the appearance of the countenance was so marked that it was almost beyond belief. The swelling seemed all to subside, and all inflammation seemed to disappear. And in an incredibly short time the face had resumed its natural appearance, except from the peeling off of the skin. The case was so severe that every hair of her head came off and she was entirely bald. Since then she has a return of a most magnificent growth of hair.

Since her recovery, in the fall of 1889, she has had no return of the symptoms.

ECZEMA.

Mrs. B. had a sore place come on the under side of the arm, three or four inches from the elbow, toward the wrist, which bore some resemblance to a carbuncle, being swollen somewhat. There was not so much pain as there was burning and itching. There were several holes from the size of a gold dollar to that of a silver quarter, filled with proud flesh ; the edges of the holes were serrated. There was a discharge of a kind of yellowish serum, but none as from a boil or an ulcer. Gradually these holes coalesced, and kept spreading until it reached the elbow and the wrist, and surrounded the arm with the exception of a strip from an inch to one and a half inches in width, extending from elbow to wrist on the upper side of the arm, of natural flesh. There would be a thick crust formation over the sore from oneeighth to one-quarter inch thick. The crust was frequently washed off with soap and water, and when thoroughly cleansed, it bore the resemblance of a crumpled piece of rose-colored satin.

On careful observation a yellow serum was seen to ooze out like drops of perspiration, which would again reform into the crust, when there would be an intense itching and irritation. This process of cleansing and encrusting continued for nearly a month or over, during which time the physician in attendance was prescribing without any result.

A portion of the crust, with pus and blood adhering, was taken from the place where it commenced, and which appeared to be the worst spot, and was sent to Dr. Swan and potentized, and the CMM in powder three times a day was given for three days. The first day there was a marked aggravation, followed on the next day by a decided relief. In the course of about a week, the improvement of the arm was very noticeable, and within a month it had almost entirely healed and become natural.

These are but two cases out of a very large number, and I suggest to my objectors to " put these in their pipes and smoke them," a common expression for careful thinking.

